# Bulk Nanostructuring of Janus‐Type Metal Electrodes

**DOI:** 10.1002/chem.202001420

**Published:** 2020-07-28

**Authors:** Dandan Gao, Si Liu, Rongji Liu, Carsten Streb

**Affiliations:** ^1^ Institute of Inorganic Chemistry I Ulm University Albert-Einstein-Allee 11 89081 Ulm Germany; ^2^ CAS Key Laboratory of Green Process and Engineering Institute of Process Engineering Chinese Academy of Sciences 100190 Beijing China; ^3^ Helmholtz-Institute Ulm Helmholtzstr. 12 89081 Ulm Germany

**Keywords:** electrocatalysis, metal oxide, nanostructure, self-assembly, surface modification

## Abstract

The stable deposition of reactive nanostructures on metal electrodes is a key process for modern technologies including energy conversion/ storage, electrocatalysis or sensing. Here a facile, scalable route is reported, which allows the bulk nanostructuring of copper foam electrodes with metal, metal oxide or metal hydroxide nanostructures. A concentration‐gradient driven synthetic approach enables the fabrication of Janus‐type electrodes where one face features Cu(OH)_2_ nanowires, while the other face features CuO nanoflowers. Thermal or chemical conversion of the nanostructured surfaces into copper oxide or copper metal is possible whilst retaining the respective nanostructure morphologies. As proof of concept, the functionalized electrodes are promising in electrocatalytic water oxidation and water reduction reactions.

## Graphical abstract

**Face‐dependent bulk nanostructuring** of Cu metal electrodes is reported, leading to Janus‐type electrodes where one face features Cu(OH)_2_ nanowires while the other face is decorated with CuO nanoflowers. Thermal conversion into Cu_*x*_O nanowires/nanoflowers as well as chemical reduction to elemental Cu nanowires/nanoflowers demonstrate the versatility of the approach. Initial electrocatalytic studies show the electrochemical reactivity of the composite electrodes.

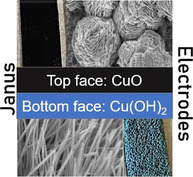

The bulk modification of metal surfaces by direct deposition of reactive nanostructures holds great promise for applications ranging from (bio‐)sensing^1^ and opto‐electronics^2^ to energy conversion and energy storage.^3^ Particularly, the in situ formation and binder‐free, stable deposition of metal oxide or metal hydroxide nanostructures has attracted significant attention, as this leads to technologically viable, scalable fabrication routes for industrially important components with relevance for sustainable energy technologies such as batteries,^4^ water electrolysis,^5^ or fuel cells.^6^ Prime examples are electrodes functionalized with copper oxide or copper hydroxide nanostructures, which have attracted widespread interest due to their use as electrocatalysts for the oxygen evolution reaction (OER)^7^ or hydrogen evolution reaction (HER),^8^ as well as supercapacitors and battery electrodes.^9^, ^10^ Consequently, a number of synthetic approaches to copper oxide/ hydroxide functionalized electrodes have been developed, including electrochemical anodization,^11^, ^12^ chemical deposition,^13^, ^14^ electrodeposition,^15^, ^16^ hydrothermal deposition^8^, ^17^ and surface oxidation.^18^, ^19^ Typically, the methods reported lead to the formation of one dominant Cu oxide/ hydroxide phase, that is, CuO, Cu_2_O or Cu(OH)_2_ with characteristic nanostructure morphologies: Cu(OH)_2_ typically forms nanowires or nanorods with diameters in the tens of nanometers and lengths of up to several micrometers. CuO often forms layered nanosheets, with individual sheet thickness of ≈100 nm. In addition, CuO “nanoflower” structures are reported where individual sheet‐like particles aggregate into spherical shapes; typical diameters of the aggregates are in the low micrometer range. Last, Cu_2_O often forms spherical nanoparticles with diameters of 50 to 150 nm.

To date, tremendous progress has been made in the bulk fabrication of phase‐pure Cu‐oxide/ hydroxide nanostructures on metal electrodes with often highly homogeneous distribution of the nanostructures across the electrode surface. In contrast, to the best of our knowledge, the fabrication of Janus‐type electrodes where the two electrode faces are functionalized with different nanostructures in one fabrication step has not been reported. This ability, however, would open new research avenues, for example, in energy conversion or sensing, where the presence of multiple, chemically distinct nanostructures enables new function. This concept has been pioneered in Janus particle research, where the functionalization of one section of a particle surface has led to ground‐breaking studies ranging from self‐propelled locomotion^20^, ^21^ to biomedical theranostics^22^ and environmental remediation.^23^, ^24^ In the context of electrodes modification, the establishment of these concepts could lead to a new multi‐component composites where several functions can be integrated within one technologically usable system.

Here, we report a facile bulk nanostructuring approach for copper foam electrodes, which enables the surface‐specific simultaneous deposition of Cu(OH)_2_ nanowires and CuO “nanoflowers” based on solution concentration gradients. Conversion of these “Janus” electrodes by thermal or chemical methods gives access to CuO or metallic Cu nanostructures which retain their original morphologies. As proof of principle, we demonstrate that the resulting composite electrodes can be used for energy‐relevant electrocatalyses, such as the oxygen evolution reaction (OER) or hydrogen evolution reaction (HER). The facile synthetic functionalization route uses the oxidation of metallic copper using ammonium peroxosulfate (APS) in aqueous sodium hydroxide solution. Immersion of a commercial copper foam (**CF**) electrode (2×10×40 mm) at room‐temperature in 15 mL of the oxidizing solution (133 mm APS, 2.66 m NaOH)—without stirring—for periods from 1 min to 15 h led to the formation of nanostructured electrodes. Note that the electrode immersed for 15 h is used hereafter and referred to as **Electrode 1**. Chemical analysis of **1** shows that the top face of the electrode (referred to as **1T**) was functionalized with black CuO, whereas the bottom face (referred to as **1B**) features blue Cu(OH)_2_, see Figure 1; for synthetic details see Supporting Information Figure S1. Scanning electron microscopy (SEM) reveals that the Cu(OH)_2_ is deposited as high aspect ratio nanowires with diameters of ≈10 nm, while the nanowire length can be tuned by variation of the immersion time, ranging from ≈2 μm (15 min immersion) to tens of micrometers (15 h immersion, Figure S2.)

**Figure 1:**
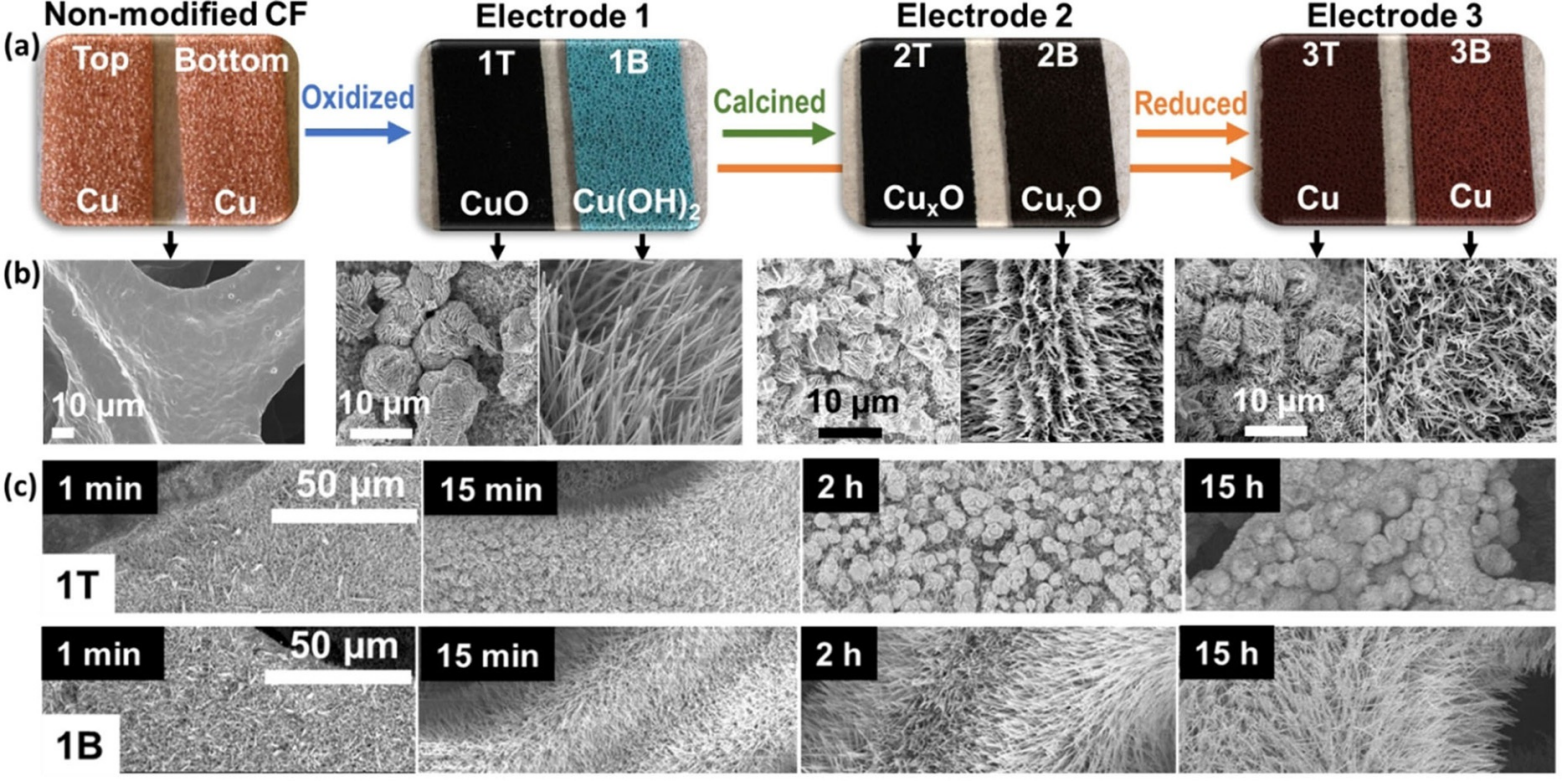
(a) Synthetic approach and photographs of the Janus‐electrodes starting from commercial, non‐modified Cu‐foam. (b) Corresponding SEM images of the electrode surfaces. (**T**: Top face. **B**: Bottom face. **1T**, **2T** and **3T**: Top face of **Electrode 1**, **2** and **3. 1B**, **2B** and **3B**: Bottom face of **Electrode 1**, **2** and **3**.) (c) Time‐dependent nanostructure growth on the top face (**1T**, CuO) and bottom face (**1B**, Cu(OH)_2_) of **Electrode** **1**.

On the CuO face, SEM analyses show the formation of spherical “nanoflower” aggregates of CuO with approximate diameters from 2 μm (15 min immersion) to 10 μm (15 h immersion, named **Electrode 1**), see Figure 1 c and Figure S2. Note that during the initial stages of nanostructuring, we also observed formation of some Cu(OH)_2_ nanowires. These disappeared upon longer immersion times; see Supporting Information, Figure S2. In addition, we noted formation of a black precipitate in the reaction vessel; this was identified as CuO, also having nanoflower morphology, see Figures S3 and S4). A cross‐section SEM analysis of **Electrode 1** clearly shows the transition from the Cu(OH)_2_ nanowires to the CuO nanoflowers and indicates that the internal part of the electrode is dominated by Cu(OH)_2_ nanowires, while the CuO nanoflowers are mainly found on the CuO face (Figure S5). Powder X‐ray diffraction (pXRD) of both electrode faces verify the structural assignments and show predominantly Cu(OH)_2_ diffraction signals for the **1B** face, while the **1T** face predominantly shows the corresponding CuO diffraction peaks; see Figure 2 a. Note that both faces show residual signals for the other nanostructure due to the porous structure of the electrode. ATR‐IR spectra (Figure S6) also indicate the structure assignments (Cu‐O‐H bending at 933 and 406 cm^−1^, Cu−O stretching modes at 600 and 471 cm^−1 [25, 26]^).

**Figure 2:**
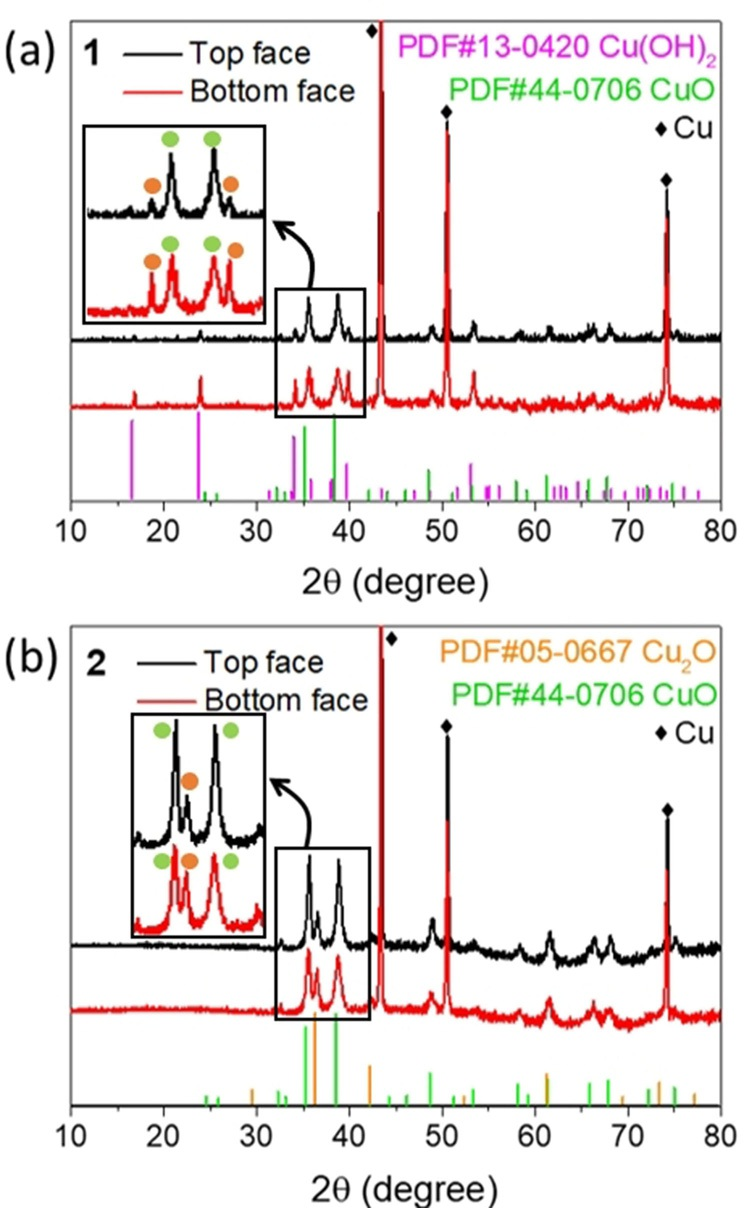
Characterization of electrodes for oxidation group. (a) XRD patterns of **Electrode 1**. (b) XRD patterns of **Electrode 2**. Inset: magnified view showing the distinct intensity differences for top and bottom face.

Conversion of the original (Cu(OH)_2_/CuO) Janus electrode into Cu_*x*_O was achieved by calcination in Ar (*T=*350 °C, *t=*1 h, named **Electrode 2**). After the treatment, both faces of the electrode were black. SEM analyses show that the original nanostructure morphologies on both faces were retained; we note that the nanowires of the **1B** face partially merge into nanosheets due to the thermal treatment, while the nanoflowers retain their structure (Figure 1 b and Figure S7). pXRD analyses of the electrode show that both faces, **2B** and **2T** feature signals for a mixture of CuO and Cu_2_O (cuprite modification); see Figure 2 b).

Next, we hypothesized that full reduction of the copper oxides to elemental Cu metal should be possible starting either from **1** or **2**. Thus, **Electrode 1** and **Electrode 2** were reacted for 2 h with aqueous NaOH solution (1 m) containing formaldehyde as reducing agent (0.4 m). This resulted in the formation of **Electrode 3**; see Figure 1 b, Figure S8 and S9. Note that virtually identical electrode morphologies were obtained independent of whether **1** or **2** were used as precursor. We observe that **3** retains the nanowire structure on the electrode bottom face (**3B**), while the nanoflower structure is retained on the top face (**3T**) (Figure S8). pXRD analysis of the samples shows that the copper oxide/hydroxide precursor nanostructures are fully reduced to elemental metallic copper (Figure S9).

As proof of concept for the electrochemical reactivity of the composite electrodes, we examined their performance on OER and HER using linear sweep voltammetry (LSV). For stability reasons we explored the Cu‐oxide/hydroxide‐containing electrodes (i.e., **Electrode** **1** and **2**) for OER and the pure Cu **Electrode** **3** for HER. Both reactions were performed in 0.1 m aqueous KOH solution at LSV scan rates of 5 mV s^−1^. All potentials are converted to the reversible hydrogen electrode (RHE) as reference. As shown in Figure 3 a, at current densities of *j*=10 mA cm^−2^ we observe overpotentials of 383 mV (**1**) and 370 mV (**2**) while significantly higher values are found for **CF** (570 mV). This means after the oxidation or calcination step, to a slightly higher overall reactivity. The Tafel slope analyses show that the functionalization of **CF** can improve the catalytic kinetics of the electrode (Figure S10). For OER, **Electrode 2** (203±0.9 mV dec^−1^) shows a lower Tafel slope than the non‐modified **CF** electrode (262±1 mV dec^−^). For HER, **Electrode 3** also shows a lower Tafel slope (344±2 mV dec^−1^) than pure **CF** (373±2 mV dec^−1^). In addition, electrochemical impedance spectroscopy (EIS) analyses (based on Nyquist plots) show, that functionalization of the electrodes **1**, **2** and **3** leads to lower charge‐transfer resistances compared with the non‐functionalized **CF** (Figure S11).

**Figure 3:**
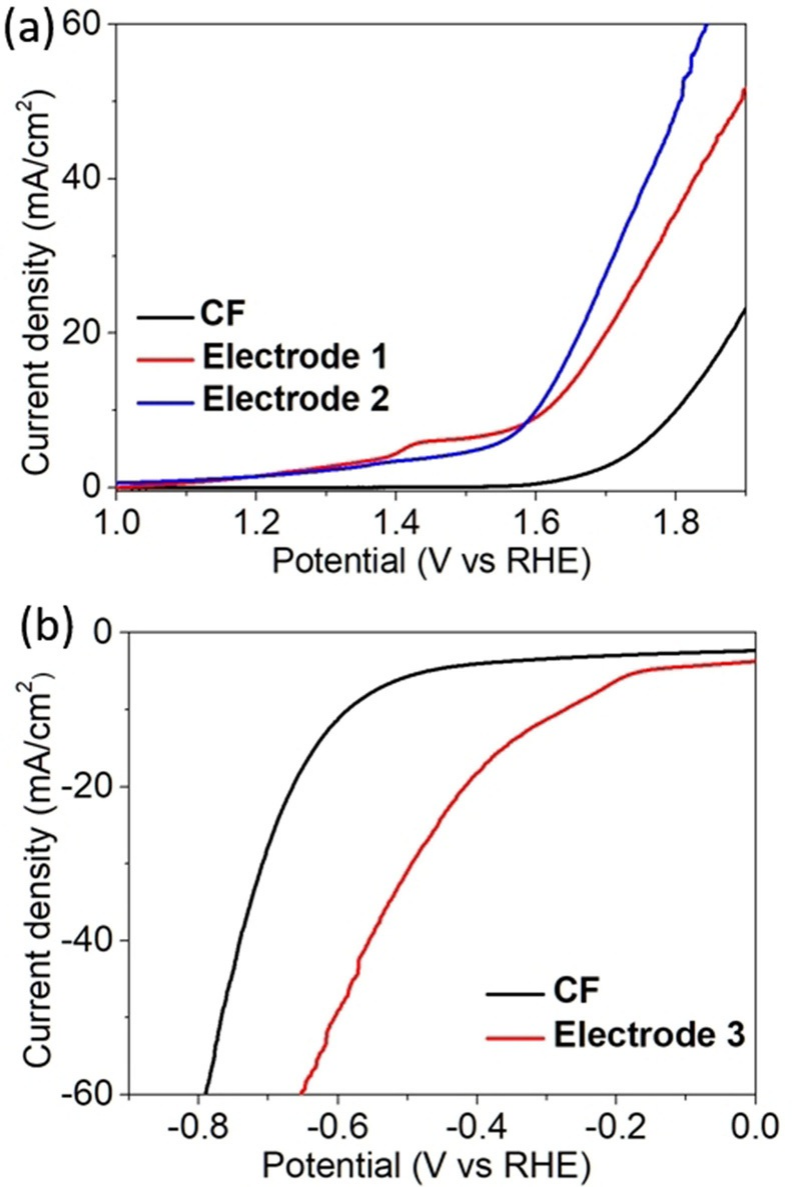
IR‐corrected polarization curves of the Cu‐functionalized electrodes. (a) OER studies of **Electrodes 1**, **2** and the reference **CF**. (b) HER studies of **3** and **CF**.

To gain insights into the unusual mechanism involved with forming different chemical structures and morphologies on the top and bottom faces of the **CF** electrode during the initial functionalization, we performed elemental analyses of the reaction solution by extracting samples from four different vertical positions (P1–P4) within the (non‐stirred) reaction mixtures (Figure 4). Analysis of the copper content of these samples using inductively coupled plasma optical emission spectroscopy (ICP‐OES) showed a Cu^2+^ gradient with higher Cu^2+^ concentrations at the bottom of the reaction vessel (Figure 4). In addition, variations of this gradient were observed depending on the reaction time, highlighting that a complex interplay between Cu^0^ to Cu^2+^ oxidation, Cu^2+^ diffusion, and Cu^2+^ precipitation (as Cu(OH)_2_ and CuO, respectively) is involved in the Janus electrode formation (Figure S12). In addition, the local OH^−^ concentration at different sites of the electrode is also a factor and will be studied in future investigations.

**Figure 4:**
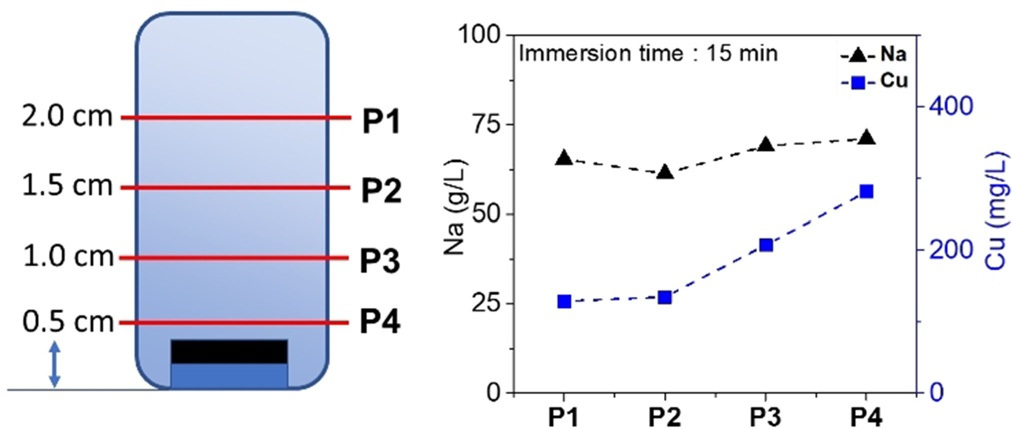
Concentration profile of Cu^2+^ and Na^+^ in the Janus electrode preparation setup.

To verify that local concentration and OH^−^ amount are the reason for the Janus electrode formation, we performed the identical electrode functionalization as described above, however, in this case, the sample was magnetically stirred. Interestingly, the resulting **Electrode 4** after 1 h immersion shows identical (black) features on both electrode faces, and the electrode surface is covered with CuO “nanoflower” aggregates (Figure S13 in Supporting Information) with a small amount of Cu(OH)_2_ detected by pXRD analysis (Figure S14). Notably however, no spatially separated nanostructures or large amounts of nanowires are observed on this electrode, thus highlighting that the proposed concentration/ pH gradient mechanism is at the bottom of the Janus electrode formation.

Finally, we compared the OER‐performance of Janus **Electrode** **1** with the non‐Janus **Electrode** **4** and observed significantly higher performance for the Janus electrode, highlighting that this facile nano/microstructuring leads to improved catalytic performance (Figure S15).

In sum, we report the first example of the facile one‐step fabrication of a nanostructured Janus‐type electrode, where simultaneously, two types of metal oxide/ hydroxide nanostructures are deposited homogeneously on a metal foam electrode. The deposition and stable attachment of Cu(OH)_2_ nanowires and CuO nanoflowers on a copper foam electrode allows their subsequent conversion into pure copper oxide nanostructures while retaining their original morphologies; chemical reduction of the copper oxides into elemental copper is also possible, again the nanostructure morphologies are retained. The resulting family of modified electrodes enable proof of concept studies for their use in electrocatalytic oxygen evolution and hydrogen evolution reactions. In future, this simple concept could be used for the fabrication of nanostructured metal oxide surface coatings with applications in sensing, energy technologies and catalysis.

## Conflict of interest

The authors declare no conflict of interest.

## Supporting information

As a service to our authors and readers, this journal provides supporting information supplied by the authors. Such materials are peer reviewed and may be re‐organized for online delivery, but are not copy‐edited or typeset. Technical support issues arising from supporting information (other than missing files) should be addressed to the authors.

Supplementary
