# Supplementary material for: Bulk Nanostructuring of Janus‐Type Metal Electrodes
Source: Chemistry. 2020 Jul 28;26(49):11109–12. doi: 10.1002/chem.202001420 (PMC7496354; doi:10.1002/chem.202001420)
Supplement: Supplementary file 1 — Supplementary [file CHEM-26-11109-s001.pdf]

Chemistry–A European Journal

Supporting Information

Bulk Nanostructuring of Janus-Type Metal Electrodes

Dandan Gao,[a] Si Liu,[a] Rongji Liu,[a, b] and Carsten Streb*[a, c]

Table of Contents

1. Instrumentation and experimental methods
2. Synthetic section
3. Janus electrode
4. Conversion to Cu_xO
5. Conversion to pure elemental Cu electrode
6. Electrocatalytic studies
7. Janus electrode formation studies
8. References
9. Author Contributions

1. Instrumentation and experimental methods

Powder X-ray diffraction (XRD) patterns were recorded on a Rigaku XRD-6000 diffractometer under the following conditions: 40 kV, 40 mA, Cu Kα radiation (λ = 0.154 nm).

Scanning electron microscopy (SEM) images were obtained using a Hitachi 5200 SEM equipment.

Fourier transform infrared spectroscopy (FT-IR) was performed on a Bruker Tensor 27 equipped with a PIKE Miracle Diamond ATR unit. Signals are given as wavenumbers in cm^{-1} using the following abbreviations: vs - very strong, s - strong, m - medium, w - weak and b - broad.

Inductively coupled plasma optical emission spectrometry (ICP-OES) was performed on a Perkin Elmer Plasma 400 spectrometer. The immersion solution was dissolved in aqueous hydrochloric acid (4 M).

Electrochemical measurements were performed on a CH Instruments CHI 760E workstation in three-electrode configuration (working electrode: “Janus” electrodes, reference electrode: mercury/mercury oxide electrode (Hg/HgO), counter electrode: platinum mesh) in 30 ml 0.1 M KOH electrolyte (pH 12.8). All electrodes were pre-conditioned by 20 cyclic voltammetry sweeps (at 100 mV/s) between 0.2 V to 0.5 V *vs*. reversible hydrogen electrode (RHE) at room temperature. **Polarization curves** for oxygen evolution reaction (OER) or hydrogen evolution reaction (HER) were recorded by linear sweep voltammetry with a scan rate of 5 mV/s (with 85% iR compensation). All potentials were converted to the RHE according to the Nernst equation ($E_{RHE} = E_{Hg/HgO} + E^0_{Hg/HgO} + 0.059\ V \times pH$). Electrochemical impedance spectroscopy (EIS) was performed using A.C. impedance spectroscopy measured at open circuit voltage at frequencies from 100 kHz to 1 Hz.

Chemicals: Ammonium peroxosulfate (**APS**), (Honeywell, CAS No. 7727-54-0), sodium hydroxide (ROTH, CAS No. 1370-73-2), potassium hydroxide (Carl Roth, CAS No. 1310-58-3), Formaldehyde (25%, MERCK, CAS No. 50-00-0). Commercial Cu foam (**CF**, dimensions 2 x 10 x 40 mm^3, 90 % porosity) was purchased from Xiamen Tmax Battery Equipments Limited, China. All chemicals were used as received.

2. Synthetic section

2.1 Preparation of the CF electrode

Commercial Cu foam (**CF**, 2 x 10 x 40 mm^3) was purchased from Xiamen Tmax Battery Equipments Limited, China. The foam possesses 90% porosity with average pore diameter of *ca.* 230 µm. Before metal oxide deposition, the **CF** was washed with acetone, deionized water and ethanol, respectively (immersion time: 15 min per solvent).

2.2 Preparation of composite Cu foam electrodes

Oxidation step: 8 mmol (1.82 g) **APS** and 160 mmol (6.40 g) NaOH are dissolved in 60 ml H_2O with stirring for 4 h to get a homogeneous solution. Then 1 piece of cleaned **CF** is immersed in a beaker containing 15 ml of the oxidant solution without stirring. The samples

are immersed for periods of 1 min, 5 min, 15 min, 40 min, 2 h, 4 h and 15 h (giving the **Electrode 1**). Then, the sample is removed, rinsed with water and ethanol, dried in air. Photographs of these electrodes are shown in Fig. S1. Note that the two electrode parts shown in each photograph are the same electrode which has been cut in half to show the top and bottom faces of the electrode. The top face (**T**) is shown in each photograph on the left, the bottom face (**B**) is shown on the right.

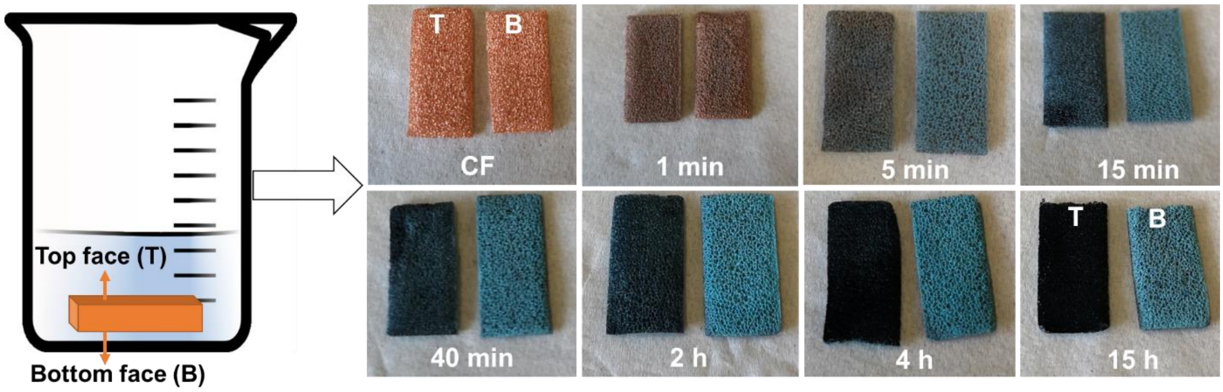

Fig. S1 Photographs of the electrodes after different immersion periods. Each photograph shows one electrode cut in half along the center. The left part is the top face of the electrode, the right part is the bottom face.

Calcination step: Calcination of **Electrode 1** is performed in a tube furnace at 350 °C for 1 h under Ar gas flow, giving the **Electrode 2**.

Reduction step: 1 ml formaldehyde (25% mass) and 20 ml 1 M aqueous NaOH are mixed and then **Electrode 1** is immersed and stirred for 2 h, giving the **Electrode 3**.

Comparison experiments: to experimentally compare the effect of stirring vs non-stirring on the Janus electrode formation, we carried out the **oxidation step** described above for pure **CF** under vigorous magnetic stirring for 1 h, giving **Electrode 4.**

3. Janus electrode

3.1: Morphological analyses of electrodes after oxidation step

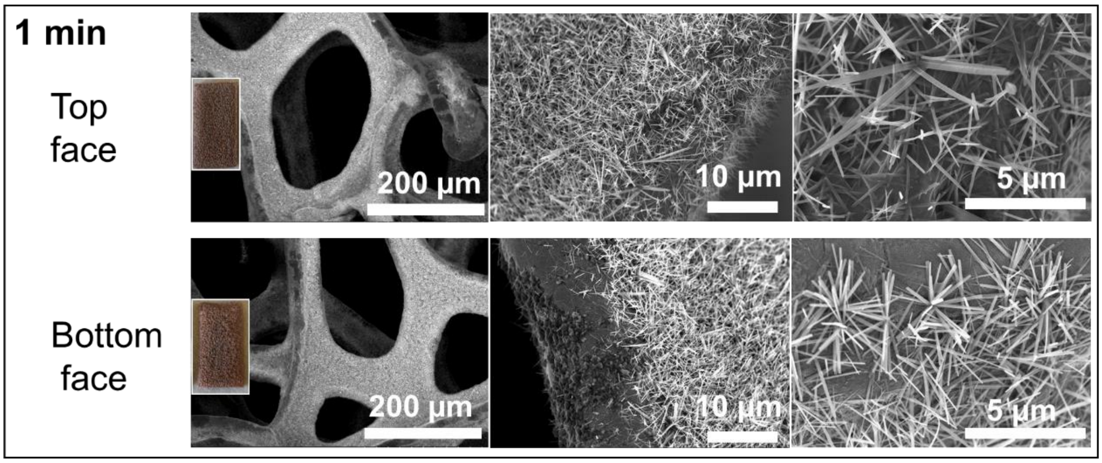

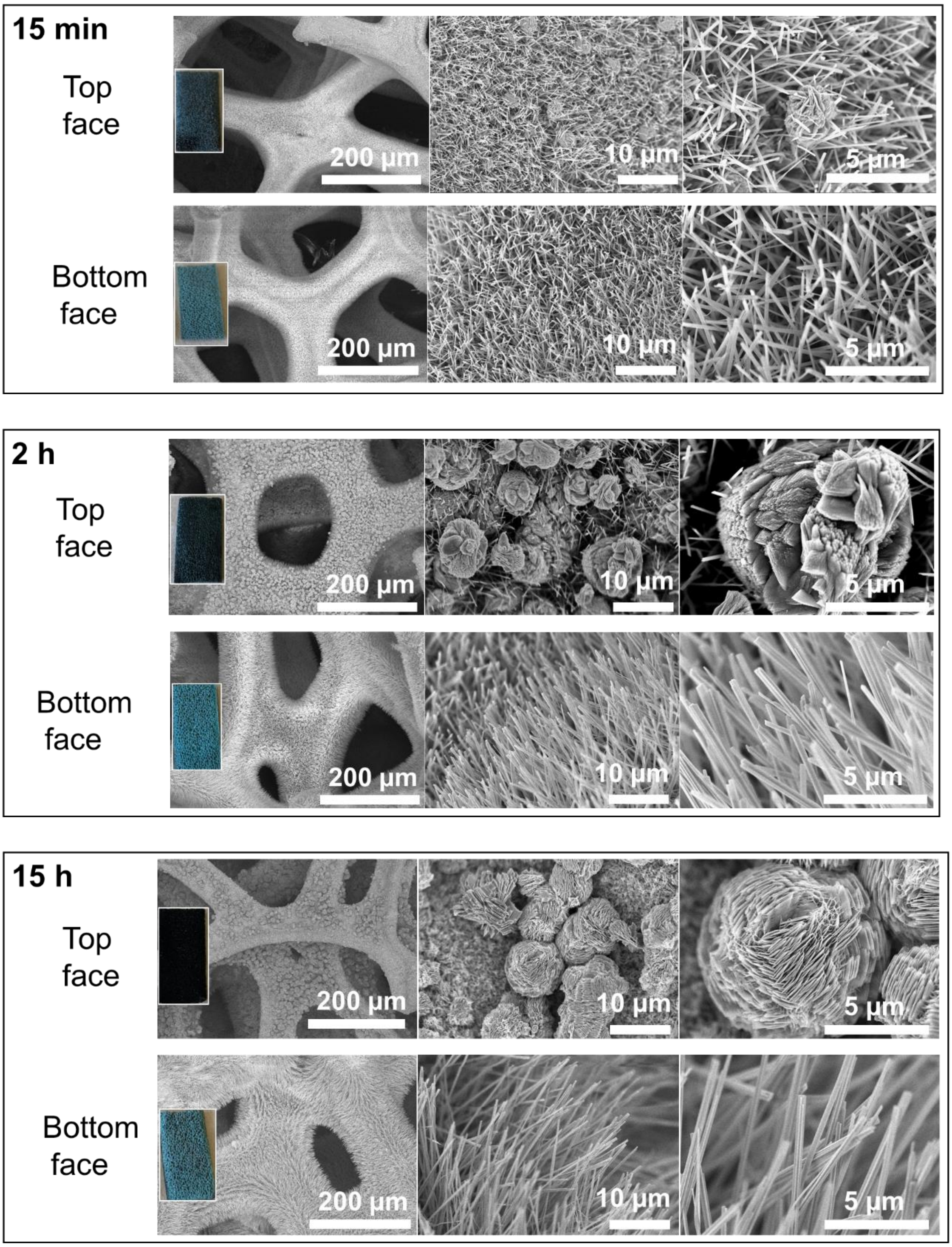

Fig. S2 Morphologies of the top and bottom faces of the oxidized **1** after different immersion periods.

3.2 Analysis of the precipitate formed during oxidation

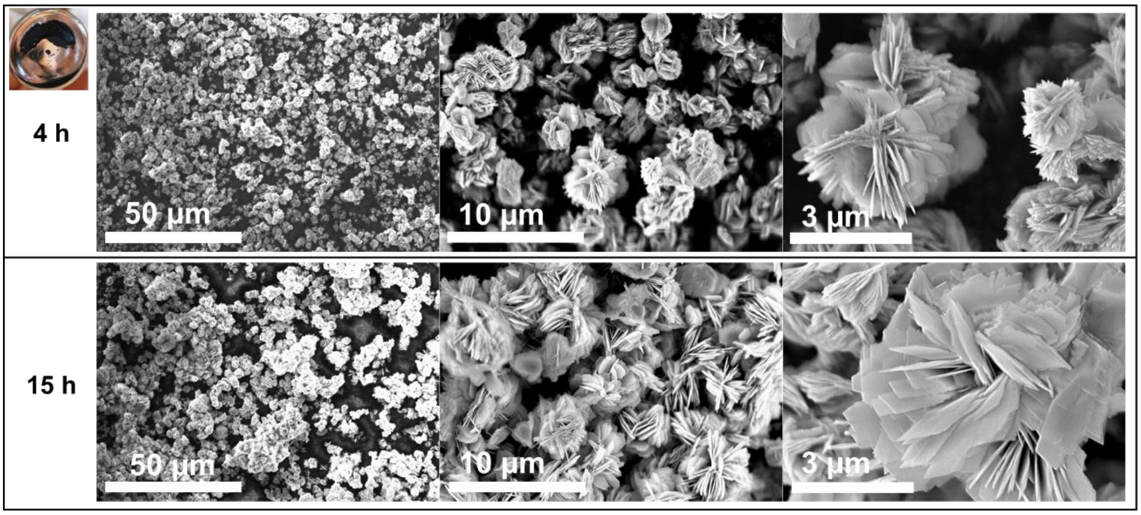

Fig. S3 SEM analysis of the precipitate collected from the bottom of reaction vessel after immersion for 4 h and 15 h.

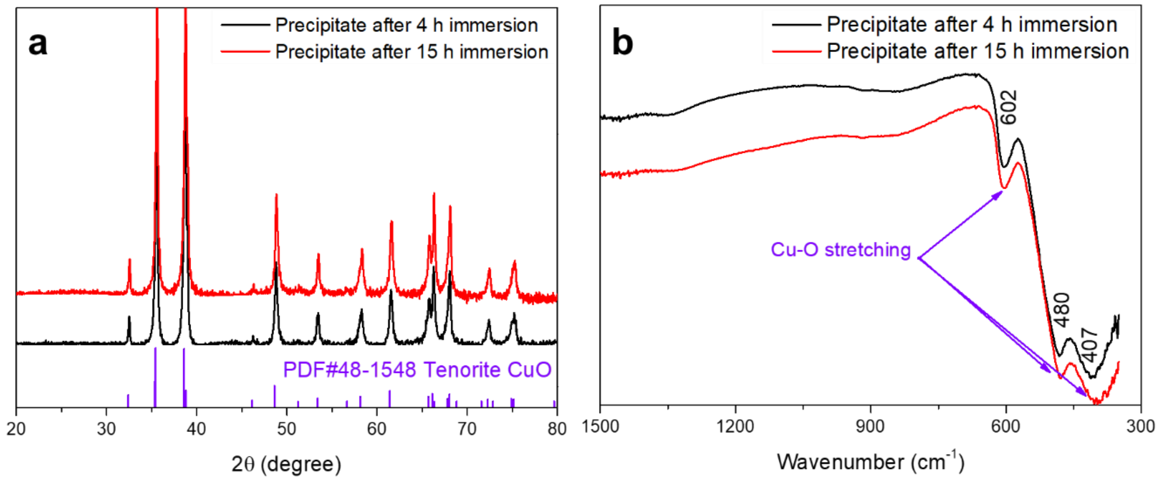

Fig. S4 pXRD patterns and IR spectrum of the precipitate collected from the bottom of the reaction flask after immersion for 4 h and 15 h.

3.3 Cross section of Electrode 1

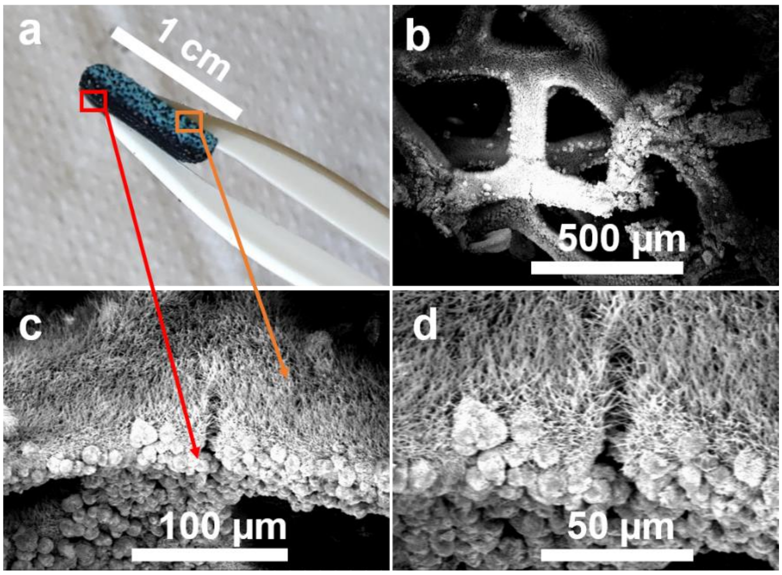

Fig. S5 SEM cross section analysis of **Electrode 1**.

3.4 IR spectra of Electrodes 1 and 2

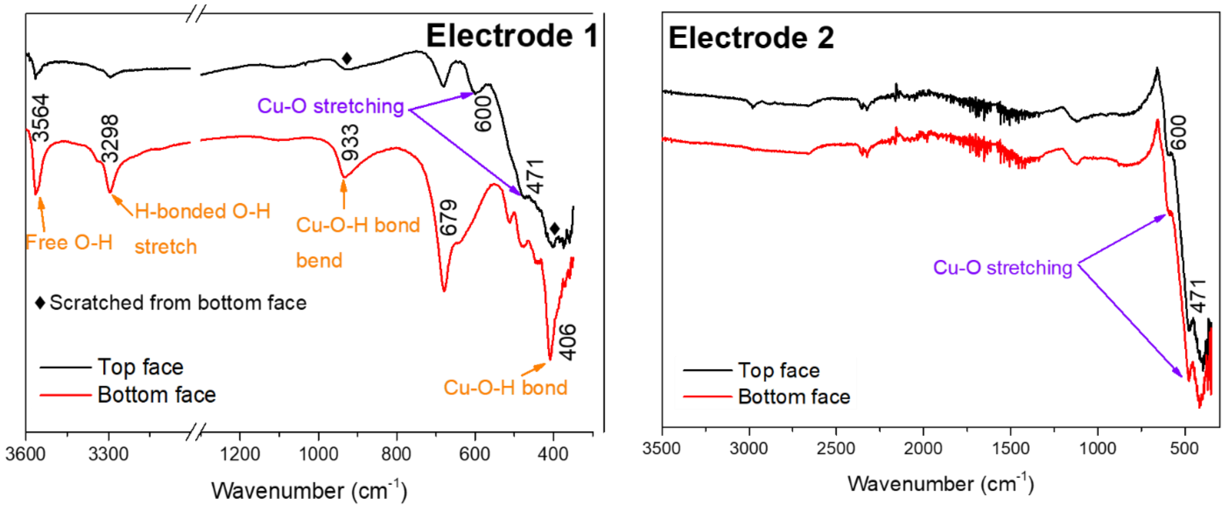

Fig. S6 ATR-IR spectra of **Electrodes 1** and **2**.

4. Conversion to Cu_xO

4.1 Morphological analyses of electrodes after calcination

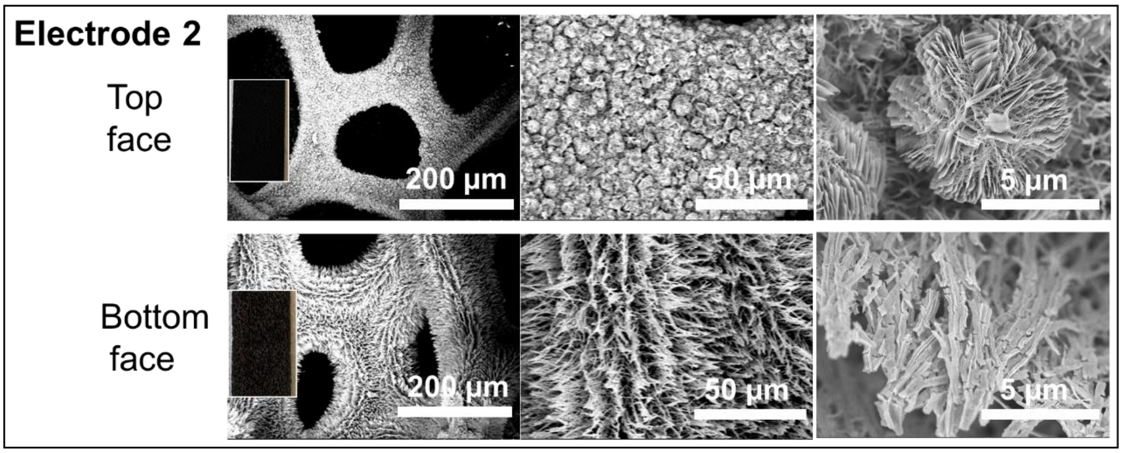

Fig. S7 SEM analysis of the top and bottom face of **Electrode 2**.

5. Conversion to pure elemental Cu electrode

5.1 Morphological analyses of electrodes after reduction

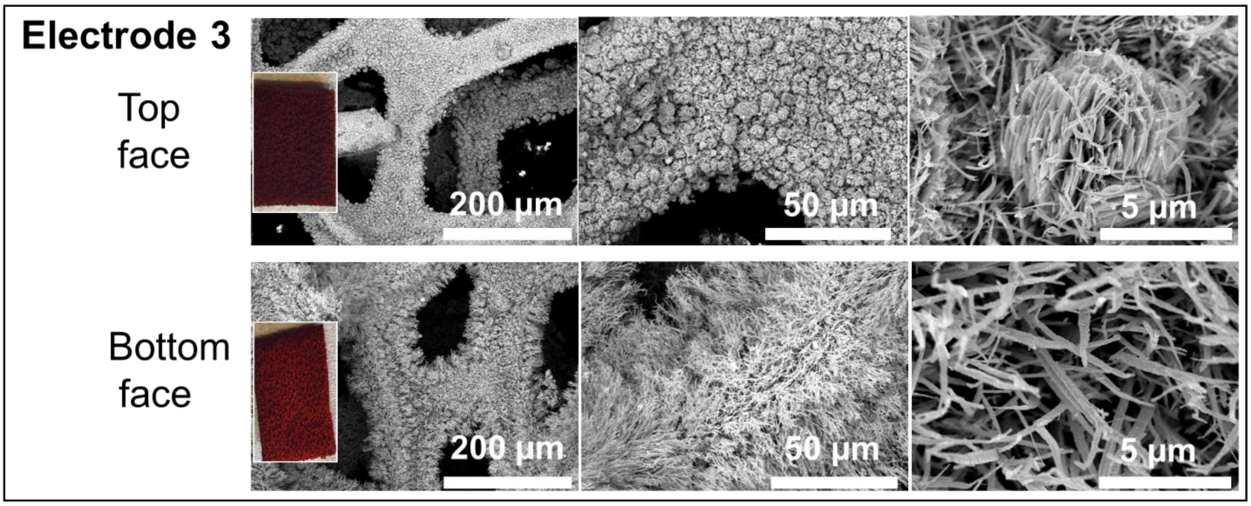

Fig. S8 SEM analysis of the top and bottom face of **Electrode 3**.

5.2 XRD patterns of electrodes after reduction

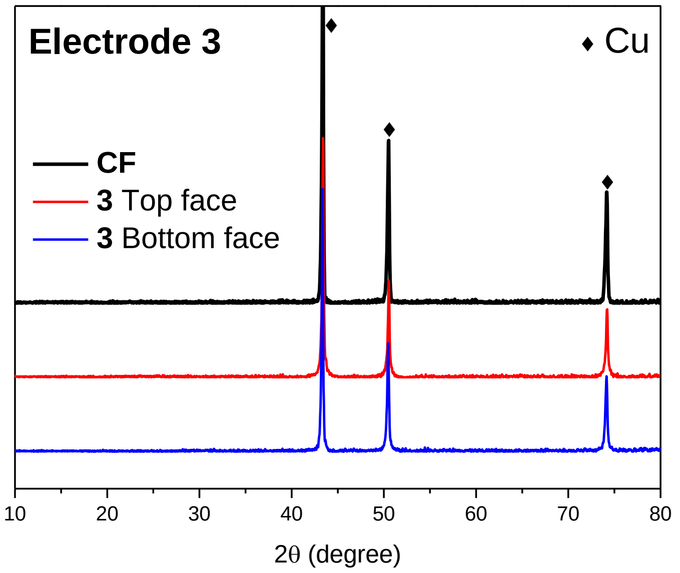

Fig. S9 pXRD patterns of the top and bottom face of **Electrode 3**.

6. Electrocatalytic studies

6.1 Exploration of catalytic kinetics

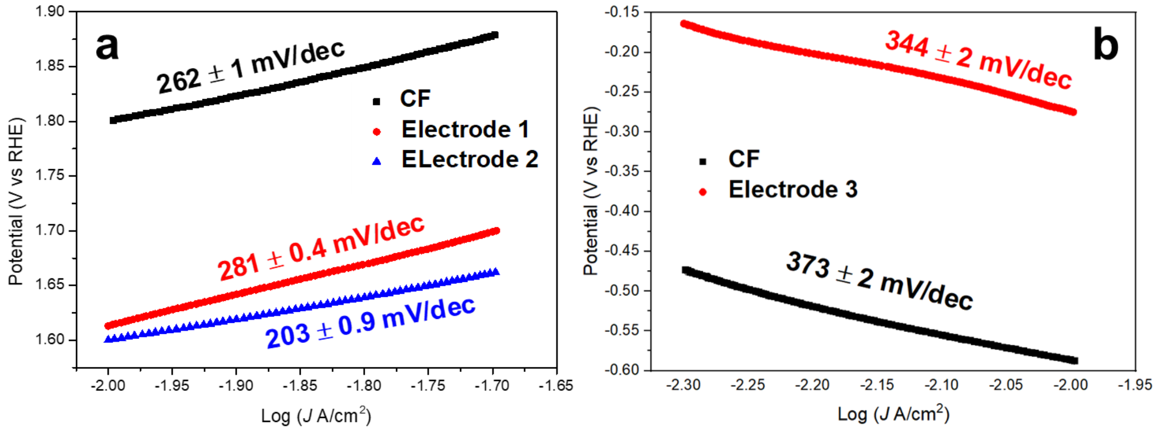

Fig. S10 OER (a) and HER (b) Tafel slopes for **Electrodes 1-3** and the pure CF.

Tafel analyses of OER by **Electrode 1** and **Electrode 2** gave Tafel slopes of 281 ± 0.4 mV/dec and 203 ± 0.9 mV/dec. For HER, **Electrode 3** shows **a** lower Tafel slope than **CF**.[1]

6.2 Electrochemical impedance spectroscopy

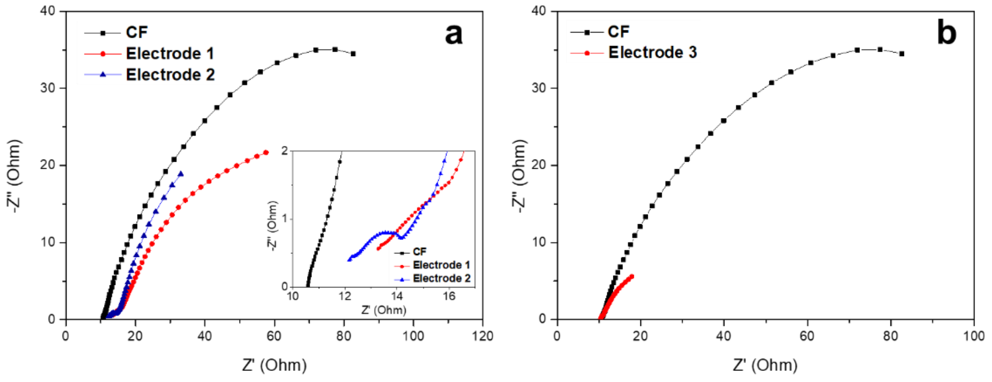

Fig. S11 Electrochemical impedance spectroscopy measured at open circuit voltage.

The Nyquist plots show, that the semicircle diameters for **Electrode 1, 2** and **3**, in the high to medium frequency region, are significantly smaller compared with non-modified **CF**.This suggests that **1**, **2** and **3** show lower charge-transfer resistances.[2]

Table S1: Comparison of electrocatalytic activity

Electrodes	Overpotential (mV) @ 10 mA/cm^2		Tafel slopes (mV/dec)	
	**OER**	**HER**	**OER**	**HER**
**CF**	570	587	262 ± 1	373 ± 2
**Electrode 1**	383	-	281 ± 0.4	
**Electrode 2**	370	-	203 ± 0.9	
**Electrode 3**	-	275		344 ± 2

7 Janus electrode formation studies

7.1 Gradients concentration of immersion solution

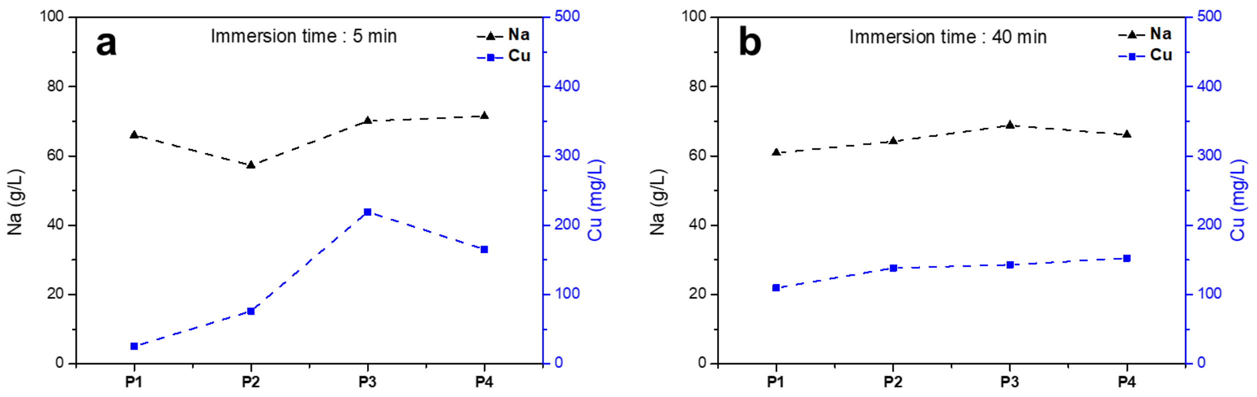

Fig. S12 ICP analysis of different positions depending on the reaction time.

7.2 Characterization of uniform electrode immersion under stirring

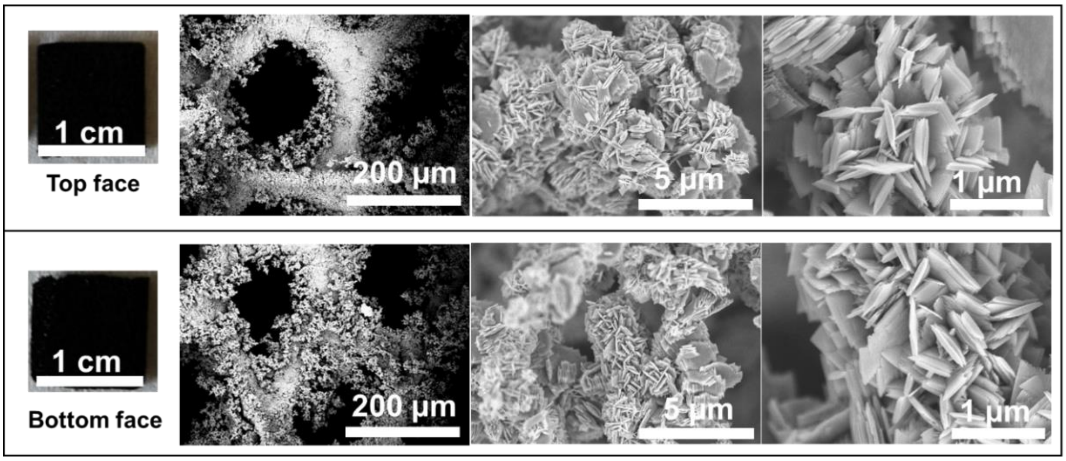

Fig. S13 SEM analysis of the top and bottom face of electrode prepared by **Electrode 4**

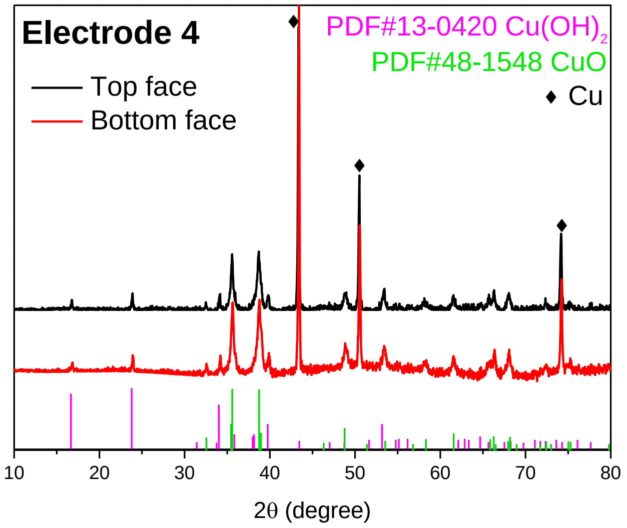

Fig. S14 pXRD patterns of the top and bottom face of the **Electrode 4**.

7.3 Comparison of OER activity of Janus electrode and uniform electrode

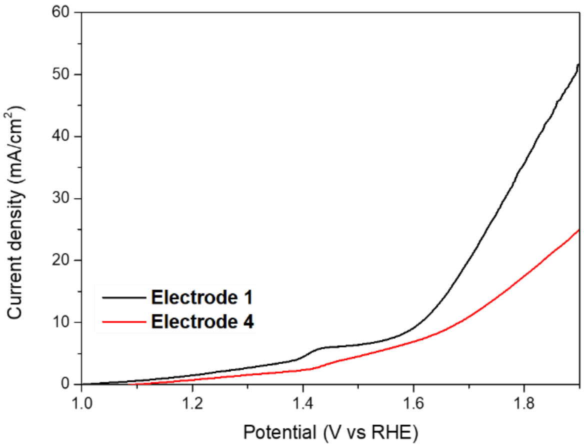

Fig. S15 IR-corrected LSV polarization curves for OER of **Electrode 1** and **4** in 0.1 M aqueous KOH, scan rate = 5 mV/s, current densities are based on geometric surface area.

According to the LSV comparison, **Electrode 4** shows lower activity and higher overpotential (453 mV @ 10 mA/cm^2) compared with the Janus **Electrode 1** (383 mV @ 10 mA/cm^2). This demonstrates that the structures present on the surface of **1** result in higher OER activity.

8. References

[1] D. Gao, R. Liu, J. Biskupek, U. Kaiser, Y.-F. Song, C. Streb, *Angew. Chem. Int. Ed.* **2019**, *58*, 4644–4648.

[2] L. He, D. Zhou, Y. Lin, R. Ge, X. Hou, X. Sun, C. Zheng, *ACS Catal.* **2018**, *8*, 3859–3864.

9. Author contributions

D.G. and C.S. conceived the idea for the project. D.G. and S.L. carried out the material design and synthesis. D.G. and R.L. evaluated the electrocatalytic performance. All authors co-wrote the manuscript.
